# *Bacillus* lipopeptides inhibit lipase activity and promote 3T3-L1 preadipocyte differentiation

**DOI:** 10.1080/14756366.2024.2417915

**Published:** 2024-10-21

**Authors:** Meichun Chen, Deju Chen, Rongfeng Xiao, Xuefang Zheng, Bo Liu, Jieping Wang

**Affiliations:** Institute of Resources, Environment and Soil Fertilizer, Fujian Academy of Agricultural Sciences, Fuzhou, China

**Keywords:** Lipopeptide, lipase inhibitory activity, 3T3-L1 adipocyte differentiation, PPARγ

## Abstract

*Bacillus* lipopeptides have been reported to display anti-obesity effects. In the present study, Lipopeptides from *Bacillus velezensis* FJAT-45028 that consisted of iturin, fengycin and surfactin were reported. The lipopeptides exhibited a strong lipase inhibition activity in a concentration-dependent manner with a half maximal inhibitory concentration of 0.012 mg/mL, and the inhibition mechanism and type were reversible and competitive, respectively. Results of CCK8 assay showed that 3T3-L1 preadipocyte cells were completely viable under treatment of 0.050–0.2 mg/mL lipopeptides for 24 or 48 h. It was found that the lipopeptides could increase lipid droplets in the differentiated 3T3-L1 adipocytes in tested concentration and suppress the expression of peroxisome proliferator-activated receptor gamma (PPARγ). These results indicated the potential anti-obesity mechanism of the tested lipopeptides might be to inhibit lipase activity but not to suppress lipid accumulation in the adipocytes. Moreover, the lipopeptides could elevate glucose utilisation by 14.43%–33.81% in the differentiated 3T3-L1 adipocytes.

## Introduction

Excessive dietary fat intake, fat accumulation and lack of exercise often lead to the appearance of obesity, which has become one of the greatest threats to human health. Obesity not only increases the risk of diseases including cardiovascular diseases, type 2 diabetes, and tumours, but also contributes to the economic burden^1^^,^^2^. Thus, to maintain a healthy body weight is of significant important. The commonly adopted treatment strategy for obesity is to use safe anti-obesity medicines combined with dietary restriction and physical exercises to achieve a normal body weight^2^^,^^3^. Currently, orlistat is the only one available anti-obesity medicine in the market, which exhibits anti-obesity effect through inhibition of lipase activity to reduce intestinal fat absorption (approximately 30% of dietary fat)^4^^,^^5^. However, orlistat was reported to cause certain gastrointestinal side effects, e.g. diarrhoea, flatulence, oily stools, oily spotting, abdominal cramping, and etc^6^^,^^7^. Hence, it is crucial to discover novel anti-obesity drugs derived from natural compound that are efficient and safe.

Natural bioactivity compounds including polyphenols, chitin, chitosan, lipstatin, panclicins and saponins derived from plants or microorganisms have been considered as potential, effective and safe anti-obesity drugs^8^^,^^9^. The mechanisms involved in anti-obesity activities of drugs are complex multi-step progress. Inhibition of lipase activity and regulation of adipogenesis are two famous mechanisms in controlling obesity^8^^,^^9^. Lipases (triacylglycerol ester hydrolases, EC 3.1.1.3) are essential enzymes for catalysing the hydrolysis of dietary triglycerides to monoglycerides and free fatty acids that are subsequently absorbed by the enterocytes cell lines in the intestine. Inhibition of lipase activity could reduce intestinal fat absorption, which is considered a valid strategy for controlling obesity^10^. It’s known that obesity is characterised at the cellular level by increasing the number of mature adipocytes and expanding the size of fat cells. Adipogenesis is a cellular differentiation process of preadipocytes into mature adipocytes and contributes to the excessive lipid accumulation^11^. Thus, to regulate adipogenesis is another therapeutic strategy for obesity. Numerous natural compounds including polyphenols and saponins are able to simultaneously inhibit pancreatic lipase activity, suppress adipogenesis and enhance fat utilisation^12^^,^^13^. Polyphenols can also modulate glucose hemostasis to display their pronounced effect on obesity^14^.

It has been reported that *Bacillus* lipopeptides could suppress lipase activity by producing inactive aqueous enzyme-surfactant complexes or by disturbing the congregation of enzymes at the lipid/water interface^15^^,^^16^. Three families (iturin, fengycin and surfactin) of lipopeptides covering about 100 chemical structural analogues produced from different *Bacillus* strains have been reported in the last two decades. Iturin/surfactin contain a *β*-amino/β-OH fatty acid linked to a peptide ring with seven amino acid residues, while fengycin has a *β*-OH fatty acid lined to a peptidic moiety with 10 amino acid residues^17^^,^^18^. It was found that iturin exerts weaker inhibition activity of lipase than fengycin and surfactin, because fengycin and surfactin could directly contact with the active amino acids of lipase yet iturin not^16^. The secretion of lipopeptide is firstly determined by the *Bacillus* species and then influenced by their culture conditions. Some strains can simultaneously generate two or three families of lipopeptides, whereas others can produce only one family^17^. For examples, *Bacillus licheniformis* strain MB01 could yield surfactin only, whereas the *B. licheniformis* strain V9T14 could co-produce surfactin and fengycin^19^^,^^20^.

Some studies have shown that the treatment with low concentration surfactants could induce differentiation of human preadipocytes^21^. *Bacillus* lipopeptides are one of the well-known biosurfactants. Regulation of the lipopeptides on adipogenesis remained to be elucidated. The 3T3-L1 preadipocytes had been extensively used as a cell culture model in studying the control of adipogenesis by anti-obesity drugs^22^. In the present study, we had obtained a *B. velezensis* FJAT-45028 strain that could co-produce three families of lipopetides. The components of the *B. velezensis* FJAT-45028 lipopeptides were identified. Then, the inhibition effect and mechanism of the lipopeptides on lipase were investigated. Finally, the influence of the lipopeptides on dipocyte differentiation was evaluated *in vitro* in the 3T3-L1 preadipocytes. This study would provide a new potential drug and more information of the lipopeptides for their application in the anti-obesity field.

## Materials and methods

### Strain, cell and reagents

Dulbecco’s Modified Eagle’s Medium (DMEM, containing 3.7 g/L Na_2_CO_3_) was purchased from Hyclone company (Logan, Utah, USA). Fetal bovine serum (FBS), bovine serum (BS) and phosphate-buffered saline (PBS) were purchased from Gibco (New York, NY, USA). Lyophilised powder of *Mucor miehei* lipase (EC3.1.1.3), 4-Nitrophenyl palmitate (4-NPP), trypsin-EDTA solution, isobutylmethylxanthine (IBMX), dexamethasone (DEXA) and insulin were purchased from Sigma-Aldrich (St. Louis, MO, USA). Trizol reagent was purchased from Invitrogen (Carlsbad, CA, USA). PrimeScript ^TM^ RT master mix for cDNA synthesis and SYBR green PCR master mix were purchased from Takara (Shiga, Japan) and Toyobo (Osaka, Japan), respectively. PPARγ ELISA kit was produced by Cayman Chemical Company, Inc. (Michigan, Colorado, USA). A154-1–1 Glucose Oxidase Activity Assay Kit was bought from Nanjing Jiancheng Biotechnology Co., Ltd, (Nanjing, China). *Bacillus velezensis* strain FJAT-45028 (CCTCC No. M 2019759) was isolated from a Mango sample and identified through whole genome sequence analyses (Gene bank number: CP047157.1). Mouse 3T3-L1 peradipocytes were purchased from Cyagen Biosciences Inc (Guangzhou, China).

### Preparation and HPLC-MS/MS analyses of the lipopeptides

The *B. velezensis* strain FJAT-45028 was cultured in media (potato extract 5 g/L, peptone 10 g/L, NaCl 5 g/L, and glucose 15 g/L) in a shaking incubator at 30 °C with a shaking speed of 170 rpm for 48 h. The cultures of FJAT-45028 were centrifuged at 6000 × g for 5 min to remove cells. The supernatants were adjusted to pH 2.0 with HCl and centrifuged at 6000 × g for 5 min to obtain the crude lipopeptides. Then, the lipopeptide extracts were dissolved in PBS and lyophilised. Finally, the lipopeptides were qualitatively and quantitatively analysed by the liquid chromatography quadrupole time-of-flight tandem mass spectrometry (LC-QTOF/MS) method according to the method described by Chen et al.^16^ The concentrations of iturin, fengycin and surfactin in the culture supernatant were calculated based on the corresponding standard curves. The concentration was defined as milligrams of standard per litre of culture supernatant (mg/L), and then the mass ratios (g/g × 100%) of each type of lipopeptides in the crude lipopeptides were further calculated.

### Lipase inhibition assay

Effect of the lipopeptides on lipase activity was evaluated by the method described by Liu et al.^23^ Firstly, 2 mg/mL of lipopeptide in water, 10 mg/mL of lipase in water and 7.5 mM of 4-NPP in acetonitrile solutions were prepared for further experiments, respectively. The furoic acid as selected as a positive control drug. The 1 mL reaction mixture contained 0.75 mM 4-NPP, 0.4 mg/mL lipase, and different concentrations of the lipopeptides in Tris-HCl buffer (pH 7.8). Then, the reaction was carried out at 37 °C using a model 1510 spectrophotometer (Thermo Fischer Scientific, Waltham, MA) at 405 nm. For determine the inhibition mechanism of lipopeptide acting on the lipase activity, the substrate concentration was fixed and the lipase concentration was changed under different concentrations of lipopeptide treatments; for determine inhibition type, the lipase concentration was fixed and the substrate concentration was changed under different concentrations of lipopeptide treatments. Then, the inhibition mechanism and type were obtained through the Lineweaver-Burk plot^23^.

### Cell viability assay

Cell viability assay was conducted to determine the potential toxicity of the lipopeptides using Cell Counting Kit-8 (CCK-8). The 3T3-L1 cells were cultured in DMEM containing 10% BS at 37 °C in an incubator with 5% CO_2_ and 95% air. Then, 100 µL of the 3T3-L1 preadipocytes were plated in a 96-well plate with a density of 1 × 10^5^ cells/well. After overnight incubation, the cells were treated with different concentrations of the lipopeptides (0.05–5 mg/mL) for 24 and 48 h. After removing the supernatant, 100 μL fresh DMEM containing 10% CCK-8 solution was added to the wells and the cells were incubated for another 2 h. Finally, the absorbance at 450 nm was measured using automatic enzyme-linked immunosorbent assay systems (SPECTRO Star). The cells treated with pure DMEM were selected as the negative control.

### Cell differentiation

The 3T3-L1 preadipocytes were inoculated into 6-well plates with DMEM media containing 10% FBS and cultured at 37 °C in a 5% CO_2_ incubator for 2 d (Day 2). The cells were differentiated with the induction media consisting of 0.05–0.15 mg/mL lipopeptides or 1.5 µg/mL rosiglitazone, 10% FBS, 0.5 mM IBMX, 1 μM DEXA and 10 μg/mL insulin in DMEM, and cultured at 37 °C in a 5% CO_2_ incubator for another 2 d (Day 4). Then, the differentiation media were replaced with 0.05–0.15 mg/mL lipopeptides or 1.5 µg/mL rosiglitazone, 10% FBS and 10 μg/mL insulin every 2 d, which continuing to the eighth day (Day 8) for cell differentiation into mature adipocyte.

### Oil red O staining

Lipid droplet accumulation in the differentiated cells was measured by Oil Red O staining. On the Day 8, the 3T3-L1 cells differentiated with or without the lipopeptides were washed with PBS and then used for the Oil Red O staining assay. Briefly, the cells were first washed twice with PBS and fixed with 4% paraformaldehyde for 15 min. After that, the cells were washed twice with PBS and then stained with Oil Red O solution for 10 min at room temperature. Subsequently, the cells were harvested and taken for microscope observing to visualise red oil droplets staining in the differentiated cells at 20× magnification. Finally, isopropanol was added into cells to harvest the free Oil Red O, and incubated at room temperature for 20 min; the absorbance of the extracted Oil Red O was measured at 520 nm using automatic enzyme-linked immunosorbent assay systems (Thermo fisher).

### Total RNA isolation and quantitative real-time PCR analysis of ppARγ1

Total RNA from the 3T3-L1 cells was extracted using Biospin Total RNA Extraction Kit (Bioer, Hangzhou, China) according to the manufacturer’s instructions. The purity was checked based on the 260:280 absorbance ratios on a Biotek Synergy H1 Multi-Mode Microplate Reader (Agilent, MA, USA). Subsequently, the cDNA was synthesised using StarScript II First-strand cDNA Synthesis Mix with gDNA Remover (Genstar, Cot#A224-10) according to the manufacturer’s protocol. The primers for the gene *ppARγ*1 (peroxisome proliferator-activated receptor gamma) were designed as: F, TGACGTGGACATCCGCAAAG; R, CTGGAAGGTGGACAGCGAGG. The gene *β*-actin (its primers were designed as: F, GACATTCAAGACAACCTGCTACA; R, CGTGTTCCGTGACAATCTGT) was used as a reference gene to normalise the results. Quantitative real-time PCR (qRT-PCR) was carried out in the ABI QuanStudio 3 real-time PCR system using SYBR Green PCR Master Mix to evaluate gene expression level. Cycling conditions were as follows: 95 °C for 5 min, followed by 40 cycles of 95 °C for 5 s, 40 cycles of 60 °C for 30s, 95 °C for 30s, 60 °C for 1 min, and 75 °C for 10 min.

### The protein PPARγ content detection

The contents of the protein PPARγ in the 3T3-L1 cells differentiation with or without the lipopeptides for 8 d were evaluated by using the PPARγ enzyme-linked immunosorbent assay (ELISA) kits according to the manufacturer’s protocol. Briefly, the differentiated 3T3-L1 adipocytes cells were collected and re-suspended with PBS. The intracellular components were extracted through repeated cycles of freezing and thawing. 50 µL of different concentrations of extracted samples and100 µL enzyme labelled reagents were added to a 96-well plate, and then the plate was incubated at 37 °C for 60 min. Subsequently, the liquid in a 96-well plate were discarded and then washed twice using a cleaning mixture. Totally 100 µL of chromogenic reagent A and B were added to the 96-well plate and the plate was incubated at 37 °C in the dark for 15 min. Then, 50 µL of terminate fluid was added to a 96-well plate to stop the reaction. Finally, the absorbance was measured at 450 nm using automatic enzyme-linked immunosorbent assay systems (Thermo fisher).

### Detection of total glucose content in the 3T3-L1 cell culture supernatant

To evaluate glucose utilisation activities of the 3T3-L1 cells in the lipopeptide-treatment group and control group, contents of the total glucose were measured in the culture supernatant after cell differentiation for 8 days using glucose oxidase activity assay kit. The culture supernatant of the cell for differentiation 8 days was collected. The working solution was consisted of phosphate buffer (100 mM), DHBS (2.0 mM), 4-Aminoantipyrine (1.0 mM), glucose oxidase 10 kU/L, MgCl_2_ (3.5 mM) and peroxidase (8 kU/L), The concentration of glucose ­standard is 5.55 mM. 2.5 µL of the sample (culture supernatant), H_2_O or glucose standard and 250 µL working solutions were added to a 96-well plate, and the plate was incubated at 37 °C for 10 min. Then, the absorbance was measured at 505 nm using automatic enzyme-linked immunosorbent assay systems (Thermo fisher). The content of glucose (mM) in the culture supernatant was calculated as = (OD_sample_ − OD_H2O_)/(OD_glucose standard_ − OD_H2O_) × 5.55 (mM) × dilution multiple.

### Statistical analysis

Significant differences among groups were considered at *p* < 0.05 by Duncan’s test using SPSS version 19.0. All the experiments were carried out in triplicate and the data values were expressed as mean ± SD.

## Results

### Component identification of the lipopeptides secreted from Bacillus velezensis FJAT-45028

**Figure 1.**
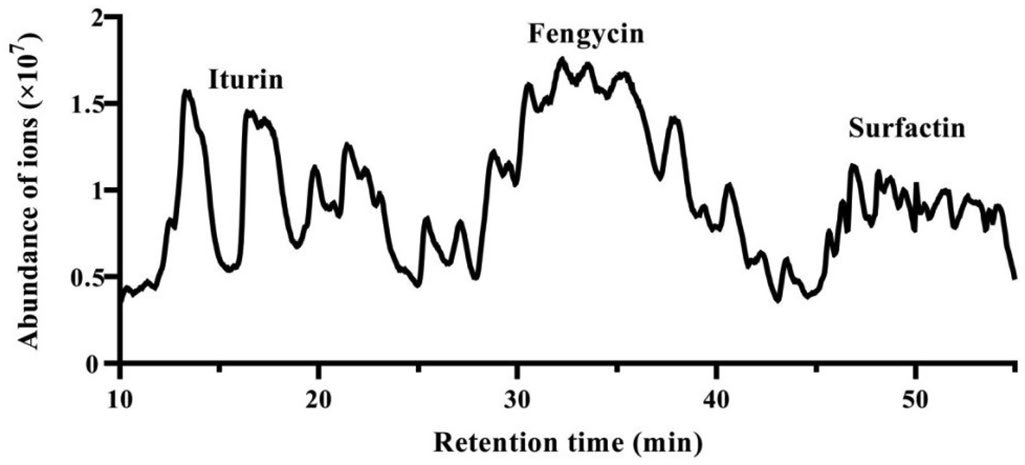
Total ion chromatogram of the crude lipopeptides from *Bacillus velezensis* FJAT-45028.

The components of the lipopeptides secreted from FJAT-45028 were analysed using LC-ESI-MS/MS technique. Three families of lipopeptides including iturins (R_t_ 13.1–26.0 min), fengycins (26.4–43.0 min), and surfactins (43.5–54.50 min) (Figure 1) were detected.

The retention times, MS and MS^2^ spectral data and identification of the FJAT-45028 lipopeptides are listed in Table 1. The results demonstrated that the lipopeptides were consisted of C_14–17_ iturin A, C_13–16_ fengycin A, C_19_ fengycin A, C_13/16_ fengycin B, C_14_ fengycin B_2_, C_16–18_ fengycin A derivative, C_12–16_ surfactin A, and C_13–14_ surfactin A derivatives (Table 1). The contents of the three components in the fermented supernatant were calculated as: 35.43 ± 2.57 mg/L for iturins, 328.56 ± 7.27 mg/L for fengycins and 99.54 ± 9.44 mg/L for surfactins, respectively. The results indicated that fengycins were the major components of the crude lipopeptides extracted from FJAT-45028.

**Table 1.** Component identification of the lipopeptides secreted from *Bacillus velezensis* FJAT-45028 using LC-QTOF-MS/MS method.

Retention time /min	MS m/z [M + H]^+^/[M + Na]^+^	MS2 m/z [M + H]^+^/[M + Na]^+^	Identification of lipopeptides
13.13	1066^a^	1037, 1020, 954, 933, 893, 795, 681, 421	C_14_Iturin A
16.24	1080^a^	1051, 1034, 1021, 968, 938, 695, 421, 285	C_15_Iturin A
17.01	1080^a^	1052, 1034, 1021, 695, 577, 497, 421, 285	C_15_Iturin A
21.31	1094^a^	1076, 1066, 1048, 1035, 1018, 823, 709, 591	C_16_Iturin A
22.89	1094^a^	1076, 1065, 1048, 1035, 1018, 983, 823, 698	C_16_Iturin A
25.42	1108^a^	1090, 1080, 1062, 1049, 997,837,421,226	C_17_Iturin A
27.05	1422^b^	1102, 988, 952, 606, 389	C_13_Fengycin A
28.81	1436^b^	1080, 966	C_14_Fengycin A
29.67	1450^b^	1103, 1094, 981, 952	C_14_Fengycin B_2_
30.67	1450^b^	1080, 966, 938	C_15_Fengycin A
31.53	1450^b^	1116, 1095, 980	C_14_Fengycin B_2_
32.29	1450^b^	1131, 1067, 952	C_13_ fengycin B
33.56	1464^b^	1080, 966	C_16_Fengycin A
34.96	1492^b^	1130, 1095, 981	C_16_ fengycin B
37.85	1506^b^	1108, 1080, 994, 966	C_19_Fengycin A
39.53	1462^b^	1080, 966	C_16_Fengycin A derivative^c^
40.70	1476^b^	1108, 1082, 994, 966	C_17_Fengycin A derivative^c^
42.33	1490^b^	1060, 966, 945	C_18_Fengycin A derivative^c^
43.60	1049^a^	936, 822, 732, 707, 661, 594, 481	C_13_surfactinA derivative
45.63	1063^a^	949, 836, 746, 707, 594, 481	C_14_ surfactinA derivative
46.40	1017^a^	903, 836.5, 790, 700, 594, 481	C_12_Surfactin A
46.90	1017^a^	904, 790, 772, 700, 594, 481	C_12_Surfactin A
48.16	1031^a^	917, 804, 714, 594, 481	C_13_Surfactin A
48.70	1031^a^	917, 804, 714, 594, 481	C_13_Surfactin A
49.47	1017^a^	917, 804, 714, 594, 481	C_12_Surfactin A
50.11	1045^a^	931, 818, 728, 594, 481	C_14_Surfactin A
50.38	1045^a^	932, 818, 728, 594, 481	C_14_Surfactin A
51.28	1045^a^	931, 818, 728, 594, 481	C_14_Surfactin A
52.55	1059^a^	945, 832, 742, 707, 594, 481	C_15_Surfactin A
53.68	1073^a^	959, 846, 756, 731, 594, 481	C_16_Surfactin A
54.22	1059^a^	946, 832, 742, 707, 594, 481	C_15_Surfactin A

^a^is [M + Na]^+^, ^b^is [M + H]^+^ and ^c^means monounsaturated β-hydroxy fatty acid.

### Effect of the lipopeptides on lipase activity

**Figure 2.**
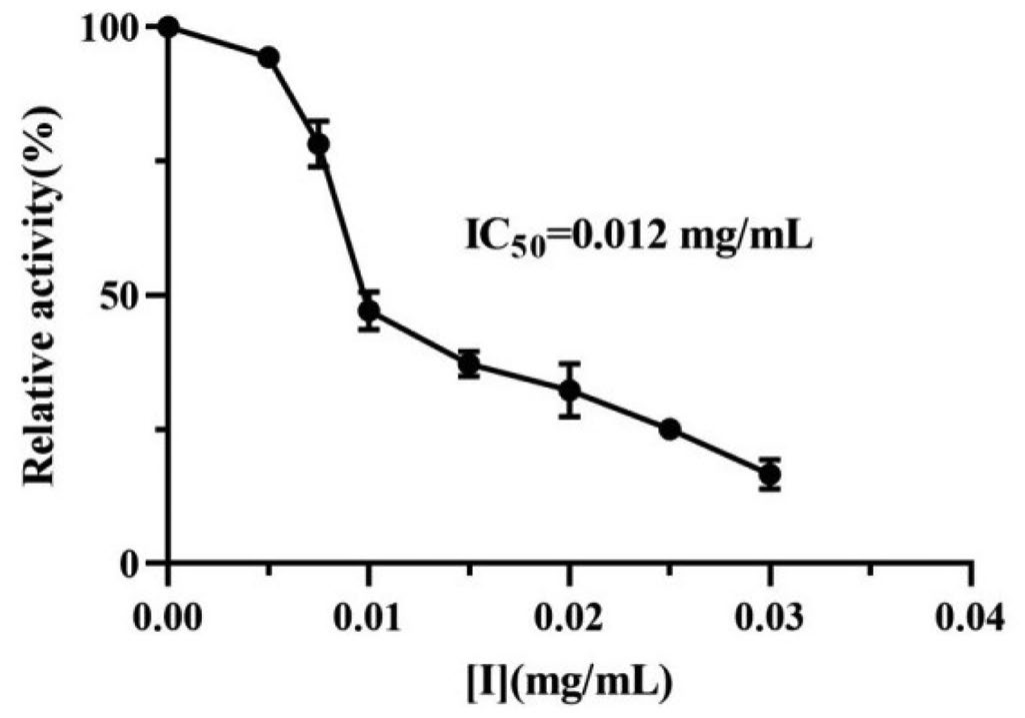
Lipase inhibitory activity of the crude lipopeptides secreted from *B. velezensis* FJAT-45028.

The anti-lipase activity of the lipopeptides secreted from *B. ­velezensis* FJAT-45028 was investigated. The results showed that the lipopeptides revealed a strong lipase inhibition activity in a concentration-dependent manner with a half maximal inhibitory concentration IC_50_ of 0.012 mg/mL (Figure 2). The IC_50_ of furoic acid was detected as 0.24 mg/mL. It was found that the IC_50_ of lipopeptide was comparable to that of orlistat (IC_50_ = 0.004 mg/mL)^24^, which suggested that the lipopeptides could act as a lipase inhibitor to prevent obesity. We had previously reported that the purified fengycin, surfactin and iturin standards inhibited lipase with IC_50_ of 0.005, 0.005, and 0.056 mg/mL, respectively^16^. The components in the FJAT-45028 lipopeptide were consisted of 7.64% iturins, 70.88% fengycins and 21.47% surfactins. Thus, the main bioactive constituents in the FJAT-45028 lipopeptides were fengycins and surfactins.

**Figure 3.**
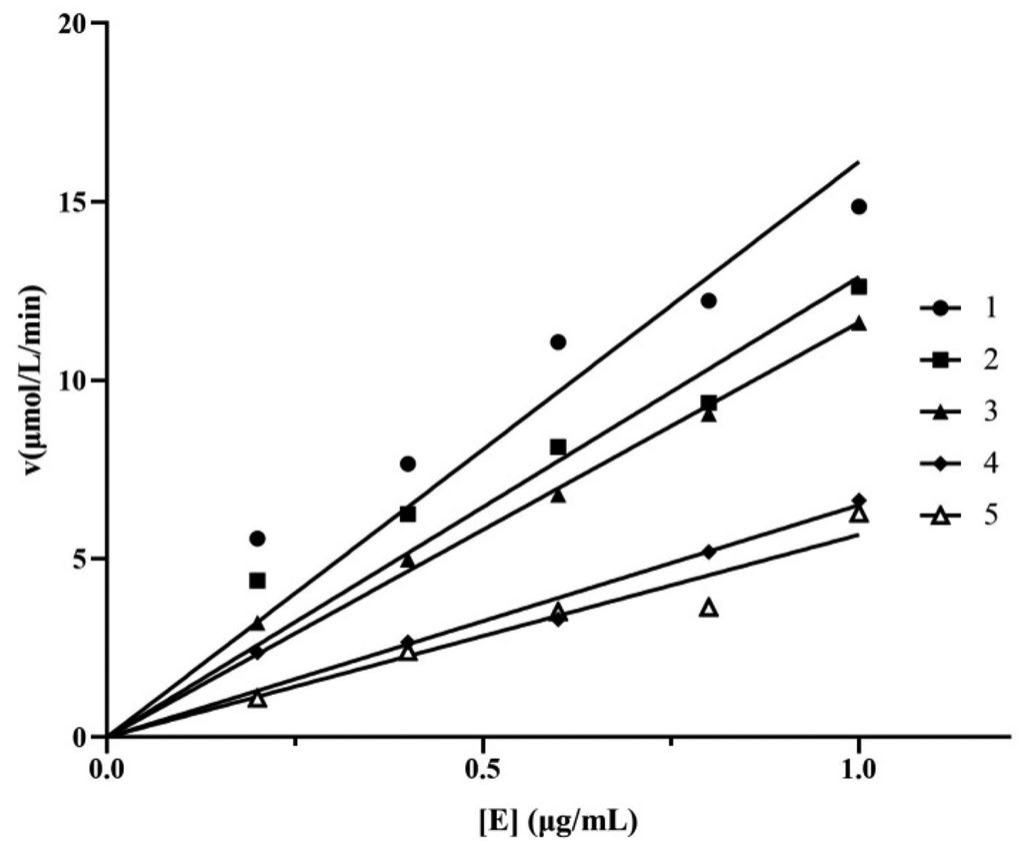
Inhibition mechanism of the crude lipopeptides, lines 1–5 correspond to different concentrations of lipopeptide: 0, 0.0013, 0.0020, 0.0025, 0.0030 mg/mL, respectively. E means different concentrations of lipase. *v* is means the initial rate of enzyme reaction.

The inhibition mechanism of the lipopeptides on lipase catalysis was studied. As shown in Figure 3, the plots of the remaining enzyme activity versus the enzyme content in the presence of varying lipopeptide concentration formed a series of straight lines that all intersected at the origin. It was found that the line slopes were declined along with the increasing concentration of the lipopeptides. The results suggested that the above catalytic action was reversible, meaning that catalytic activity of lipase was declined but not deactivate under the treatment of the lipopeptides.

**Figure 4.**
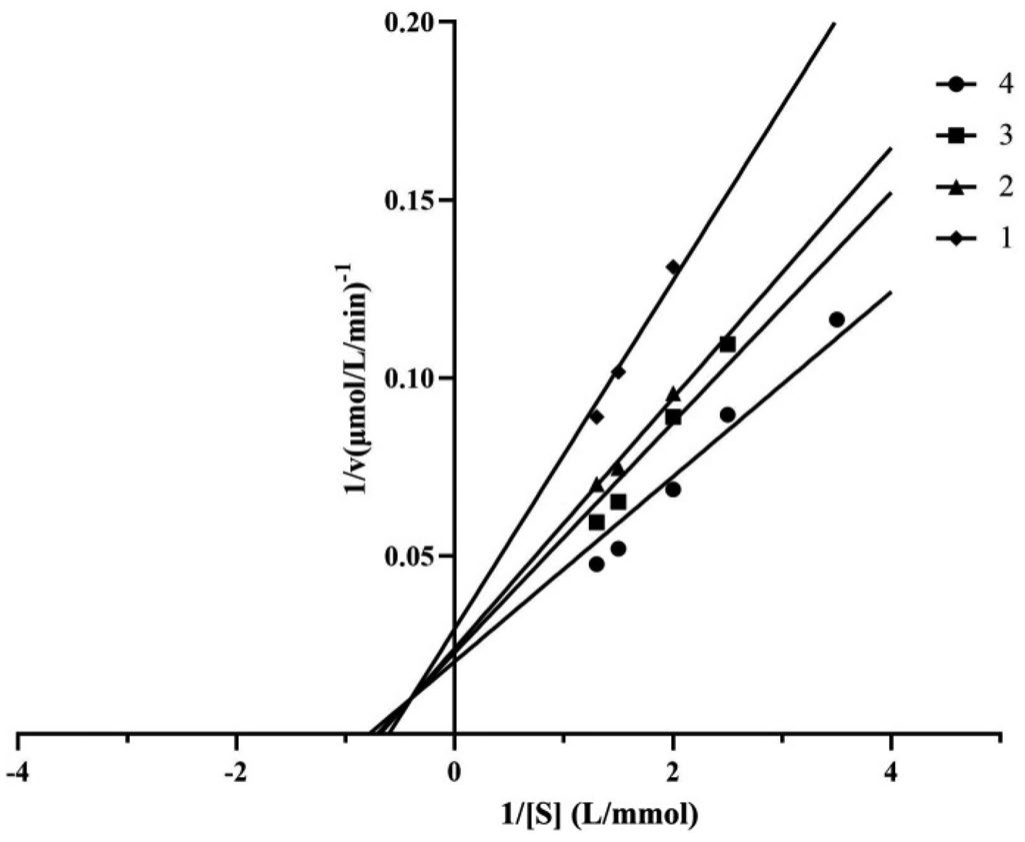
Inhibition type of the crude lipopeptides, lines 4–1 correspond to different concentrations of lipopeptide: 0, 0.0013, 0.0020, and 0.0025 mg/mL, respectively. S means different concentrations of substrate. v is means the initial rate of enzyme reaction.

To further characterise the lipase inhibitory pattern by the lipopeptides, plots of 1/*v* versus 1/[S] of the lipopeptides on lipase was drawn (Figure 4), where *v* is the reaction rate and *S* is the substrate concentration. The results revealed that the lipopeptides displayed a competitive inhibition mode on lipase activity. This finding suggested that the lipopeptides could bind to the free enzyme at the substrate-binding site.

### Effect of the lipopeptides on 3T3-L1 cell viability

**Figure 5.**
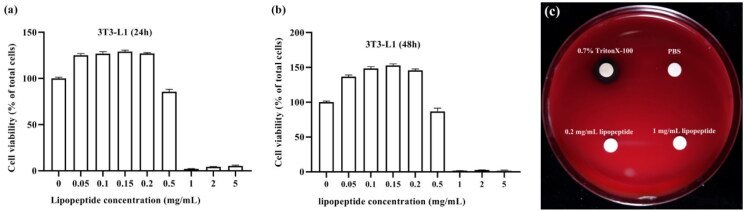
Effect of different concentrations of the lipopeptides (0–5 mg/mL) on 3T3-L1 adipocyte viability for 24 h (a) or 48 h (b), and the plate haemolytic tests of the lipopeptides at 0.2 mg/mL or 1 mg/mL, PBS was the negative control, 0.7% TritonX-100 was a positive control (c). The values were obtained from three independent experiments and expressed as the means ± SD.

To assess whether the lipopeptides with concentrations of 0.05–5 mg/mL were safe for the 3T3-L1 cells, the CCK8 assay was performed to measure cell viability. As shown in Figure 5(a,b), the lipopeptides did not affect cell viability up to 0.2 mg/mL, no matter for 24 or 48 h. However, treatment with 0.5 mg/mL of the lipopeptides induced cell damage and decreased cell viability by 15%. Based on these results, the concentrations of the lipopeptides were used in the range of 0.05–0.2 mg/mL for further studies. In addition, the plate haemolytic tests of the lipopeptides from FJAT-45028 were carried out. It was found that the lipopeptides did not display haemolytic activity even at the higher concentration (1 mg/mL, Figure 5c).

### Lipid accumulation in the 3T3-L1 adipocytes induced by the lipopeptides

**Figure 6.**
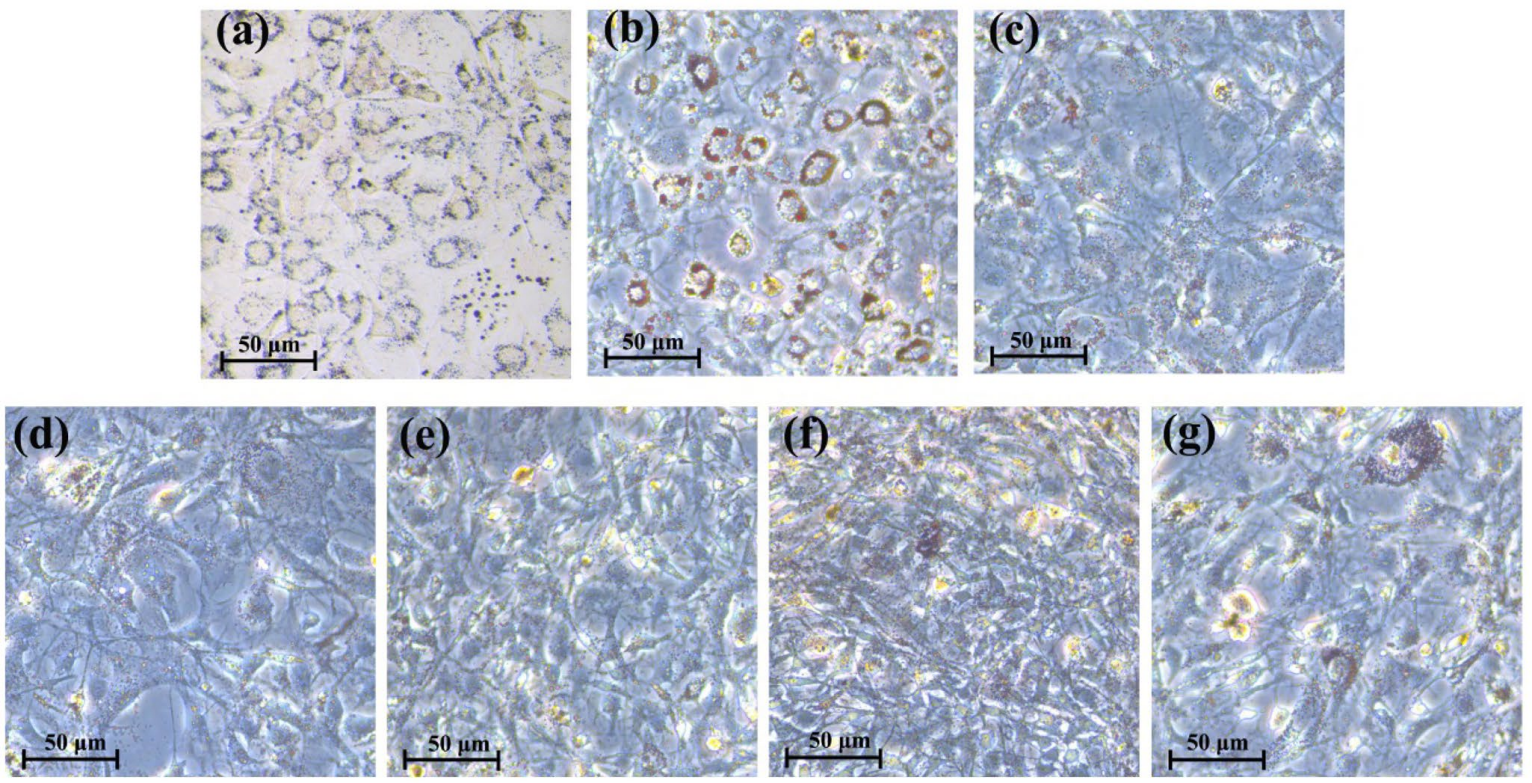
Oil Red O staining of the differentiated 3T3-L1 adipocytes. (scale bar = 50 μm). (a) negative group (H_2_O-treated group); (b) positive group (1.5 µg/mL rosiglitazone); (c–g) lipopeptide-treated group (50, 75, 100, 125 and 150 µg/mL, respectively).

Lipid accumulation is a crucial indicator of adipogenesis. The effects of the lipopeptides and rosiglitazone on the storage of intracellular lipid in the mature 3T3-L1 adipocytes were visualised by Oil Red O staining as shown in Figure 6.

**Figure 7.**
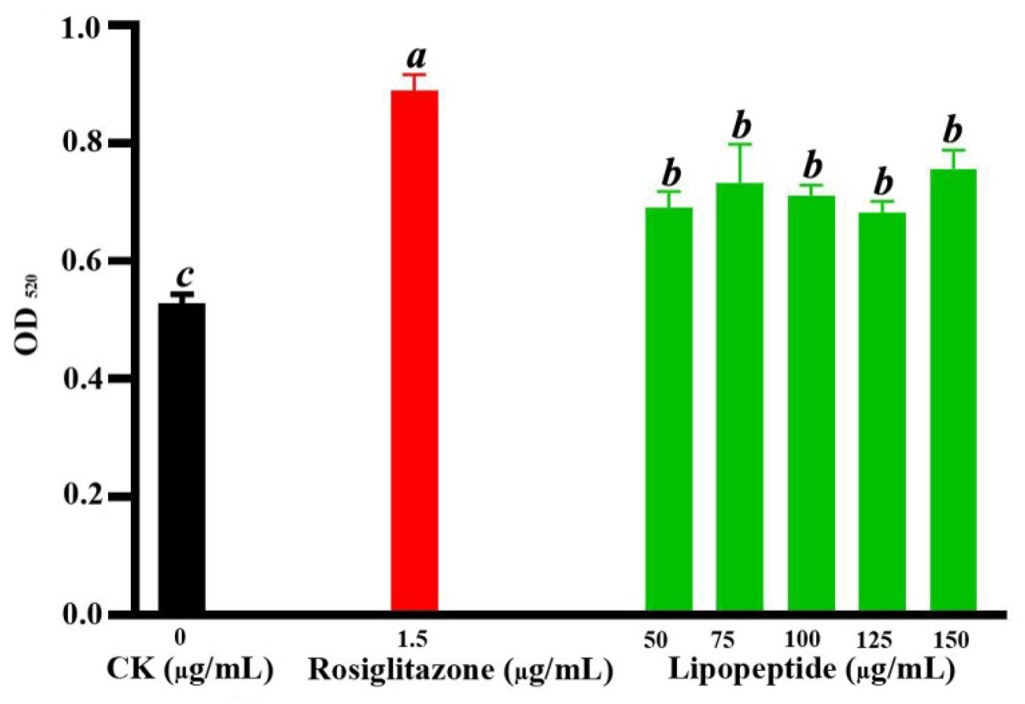
The Oil Red O contents of cells under the treatments of CK (negative control group), rosiglitazone (1.5 µg/mL) and different concentrations of lipopeptides (50, 75, 100, 125 and 150 µg/mL). The difference letter indicated that the differences among the samples are significant through the Duncan test (*p* < 0.05).

The results showed that the lipopeptides and rosiglitazone did not suppress the oil droplet accumulation of the 3T3-L1 adipocytes. Furthermore, the amount of oil red O in lipid ­droplets was measured at 520 nm using a microplate reader. Comparing to the negative control cells, the Oil Red O contents were increased by 15%–30% and 31% in the 3T3-L1 adipocytes treated by the lipopeptides and rosiglitazone, respectively (Figure 7). In addition, it was found that lipid accumulation had no significant difference in the cells treated with different concentration of the lipopeptides. The above data indicated that the lipopeptides could promote differentiation of the 3T3-L1 adipocytes and increase number of the oil droplet in a concentration range of 50–150 µg/mL.

### Effect of the lipopeptides on expressions of PPARγ at transcriptional and translational levels

Adipogenesis involves the differentiation of preadipocytes into adipocytes and lipid accumulation, which is mediated by the expression of various transcription factors and adipogenesis related genes. Among them, the PPARγ is a marker of adipogenesis. The results of RT-qPCR demonstrated that transcriptional activity of the gene *ppARγ* in the 3T3-L1 cells treated with the lipopeptides was significantly down-regulated during adipocytic differentiation (Table 2). Furthermore, the results of ELISA showed that the expression level of the protein PPARγ was also inhibited in the 3T3-L1 cells by the lipopeptides (Table 2).

**Table 2.** Expression levels of the gene *ppARγ*1 and the protein PPARγ.

		Control	Rosiglitazone 1.5µg/mL	Lipopeptide (µg/mL)				
				50	75	100	125	150
Gene 2^−ΔΔct^	*ppAR*γ1	1*^b^*	1.36 ± 0.18*^a^*	0.48 ± 0.04*^c^*	0.36 ± 0.03 *^cd^*	0.43 ± 0.01*^c^*	0.44 ± 0.02*^c^*	0.30 ± 0.02*^d^*
Protein (ng/mL)	PPARγ	1.85 ± 0.01*^a^*	1.73 ± 0.01*^c^*	1.79 ± 0.01*^b^*	1.36 ± 0.01*^e^*	1.74 ± 0.01*^c^*	1.78 ± 0.01*^b^*	1.51 ± 0.01*^d^*

The difference letter in the same line indicated that the differences among the samples are significant through the Duncan test (*p* < 0.05).

### 3.6. Effect of the lipopeptides on glucose utilisation during 3T3-L1 cell differentiation

**Figure 8.**
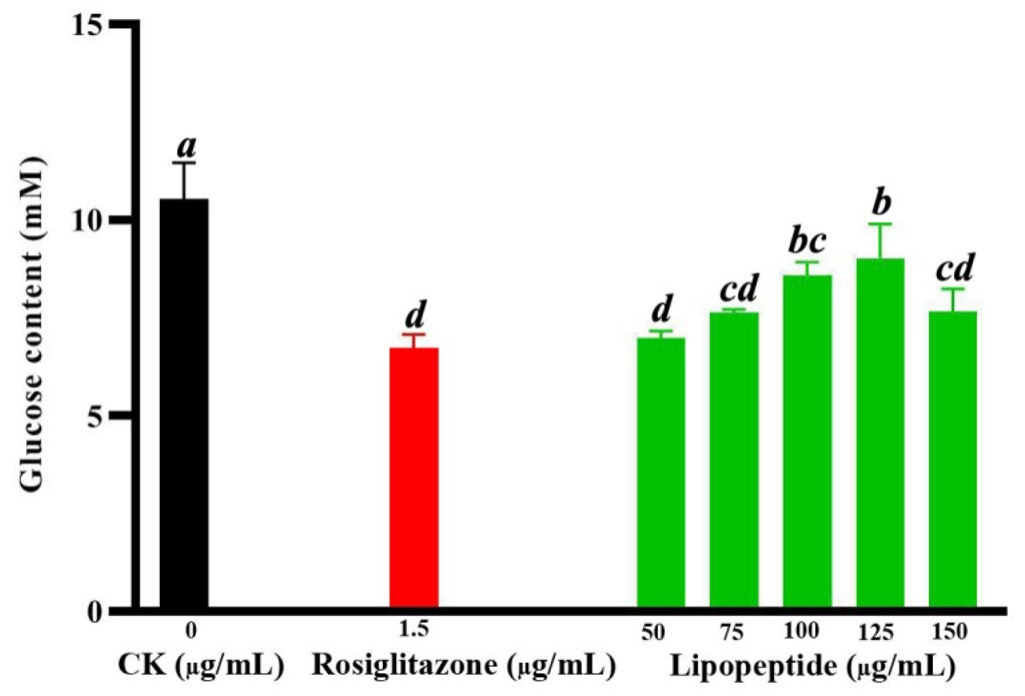
The contents of glucose in the culture supernatant after cell differentiation for 8 d. The difference letter indicated that the differences among the samples are significant through the Duncan test (*p* < 0.05).

Interestingly, it was found that the glucose utilisation during 3T3-L1 cell differentiation was significantly elevated by 14.43%–33.81% in the treatments of 50–150 µg/mL lipopeptides, comparing to the negative control cells (Figure 8). Meanwhile, a known commercial hypoglycaemic drug rosiglitazone (1.5 µg/mL) could enhance the glucose utilisation by 36.09% (Figure 8). The results indicated that the FJAT-45028 lipopeptides would have a potential to be a hypoglycaemic drug.

## Discussion

Microbiological origin compounds acting as lipase inhibitors have been considered as potential anti-obesity drugs, such as lipstatin isolated from *Streptomyces toxytricini*^25^, panclicins A-E isolated from *Streptomyces* sp. nr 0619^26^, ebelactones A/B derived from *Streptomyces aburaviensis*^27^, obafluorin produced by *Pseudomonas fluorescens*^28^, and vibralactone produced by *Boreostereum vibrans*^29^. These compounds exhibiting their lipase inhibition activities depended not only on the presence of *β*-lactone ring, but also the chemical structure of amino acid moiety and alkyl chain that bring about amphiphilic character of the molecule and in potential aggregation at lipid/water interface^25^. The famous anti-obesity drug orlistat is known as the hydrogenated form of lipstatin, which prevents hydrolysis of triglycerides by inhibiting the activities of pancreatic and gastric lipases, thereby reducing their absorption and the amount of free fatty acids and monoglycerides in the intestine^30^.

Surfactants were reported to affect lipase activity in a complex mode by forming a shield that hinders the access of lipase to the oil/water interface and increasing the solubility of the substrate that may enhance the lipolytic action of the enzyme^15^. The lipopeptides secreted from the *Bacillus* strains are a type of famous surfactants and have lipase inhibition activity. For example, Chen et al., demonstrated that the crude lipopeptides from strain FJAT-52631 inhibited lipase with IC_50_ of 0.011 mg/mL by direct interact with the active site of lipase^16^. In this study, the crude lipopeptides produced from *B. velezensis* FJAT-45028 exhibited an inhibition activity of lipase with IC_50_ of 0.012 mg/mL, being identical to the FJAT-52631 lipopeptides. However, the lipopeptide yield in the culture supernatant of the strain FJAT-45028 (463.53 mg/L) was 3.7-fold that of the strain FJAT-52631 (125.94 mg/L). These results suggested that the strain FJAT-45028 was a good candidate for producing lipopeptides with strong lipase inhibition activity.

Four inhibition types including competitive, non-competitive, uncompetitive, and mixed type have been suggested for enzyme inhibitors, which are affected by the structure characters and molecular weight of inhibitors^31^. Our results showed that the FJAT-45028 lipopeptides repress lipase activity in a in a competitive type, which is different to the result reported by Zouari et al, who found that the *B. subtilis* SPB1 lipopeptides suppressed the lipase activity via uncompetitive manner^32^. In generally, the enzyme inhibition mechanism of drug is reported as reversible or irreversible. For examples, furoic acid and oxalic acid exhibited reversible effects, while orlistat displayed irreversible action^23^^,^^33^. In the present study, the FJAT-45028 lipopeptides exhibited a reversible enzyme inhibition mechanism on lipase activity.

Besides inhibiting lipase activity, lipase inhibitors from nature-source could also exhibit anti-obesity effects via down-regulating the lipid accumulation^34^. The excess accumulation of lipids in the adipose tissue leads to obesity, which is caused by both cellular hyperplasia and hypertrophy^8^^,^^9^. Hyperplasia occurs during the adipogenesis process that differentiates preadipocytes into mature adipocytes. Surfactants in low concentration have been reported to induce differentiation of human preadipocytes^21^. In present study, we also found that the lipopeptides secreted from FJAT-45028 could induce adipocyte differentiation in 3T3-L1 preadipocytes and increase the lipid droplet accumulation in the tested concentration range of 50–150 µg/mL. These findings suggested that the potential anti-obesity effects of the tested lipopeptides might be to inhibit lipase activity but not to suppress lipid accumulation in the adipocytes.

The transcriptional regulators including C/EBP family (Cytosine-Cytosine-Adenosine-Adenosine-Thymidine/Enhancer-binding proteins) and PPARγ are considered to regulate the differentiation of preadipocytes at the early stage in mammalian cells^35^^,^^36^. It was also found that rosiglitazone could significantly increase lipid droplets and up-regulate the expression of the gene *ppARγ* in 3T3-L1 adipocytes, which was consistent with the previous studies that rosiglitazone stimulates adipocyte differentiation via the activation of PPARγ^37^^,^^38^. Previous studies showed that many natural products could down-regulate both mRNA and protein levels of PPARγ to inhibit differentiation and lipid accumulation in 3T3-L1 adipocytes^34^. It was unexpected that the tested lipopeptides (50–150 µg/mL) decreased expression of PPARγ at both transcriptional and translational levels, but induced lipid accumulation during the differentiation process of the 3T3-L1 adipocytes in a dose-independent manner. Taken together, these results suggested that the action mechanism of lipopeptide-induced 3T3-L1 adipocyte differentiation might be a PPAR γ-independent pathway.

Many literatures reported that the adipogenic related genes are mainly associated with cellular uptake of glucose and fatty acid, triglyceride hydrolysis or lipogenesis^39^. Our results showed that the lipopeptides repressed the expression of adipogenic-related gene *ppARγ* yet improved 3T3-L1 cellular glucose utilisation. The results were similar to the previous reports that the compound BL6 decreased expression of adipogenic genes and increased glucose uptake by 3T3-L1 cells^40^. Rosiglitazone is a commonly used antidiabetic drug, which has been reported to significantly enhance glucose uptake into muscle and adipose tissue^41^. The tested lipopeptides displayed the similar effect of rosiglitazone in promoting glucose utilisation.

## Conclusions

The lipopeptides from *B. velezensis* FJAT-45028 exhibited a strong lipase inhibition activity with reversible and competitive action modes. The lipopeptides could induce 3T3-L1 preadipocyte differentiation in a PPARγ-independent pathway and promote glucose utilisation in the differentiated 3T3-L1 adipocytes. The lipopeptides could be selected as health dietary supplements due to their strongly inhibiting lipase activity and promoting glucose utilisation ability. Thus, it’s worthwhile to further investigate the pharmacological effects of the lipopeptides.

## Supplementary Material

Original Image for Fig 6c.jpg

Original Image for Fig 6e.jpg

Original Image for Fig 6d.jpg

Original Image for Fig 6a.jpg

Original Image for Fig 6g.jpg

Original Image for Fig 6f.jpg

Original Image for Fig 6b.jpg

## Data Availability

The data underlying this article are available in the article; further inquiries can be directed to the corresponding author.
