# Supplementary figures and images for: Bacillus lipopeptides inhibit lipase activity and promote 3T3-L1 preadipocyte differentiation
Source: J Enzyme Inhib Med Chem. 2024 Oct 21;39(1):2417915. doi: 10.1080/14756366.2024.2417915 (PMC11497581; doi:10.1080/14756366.2024.2417915)

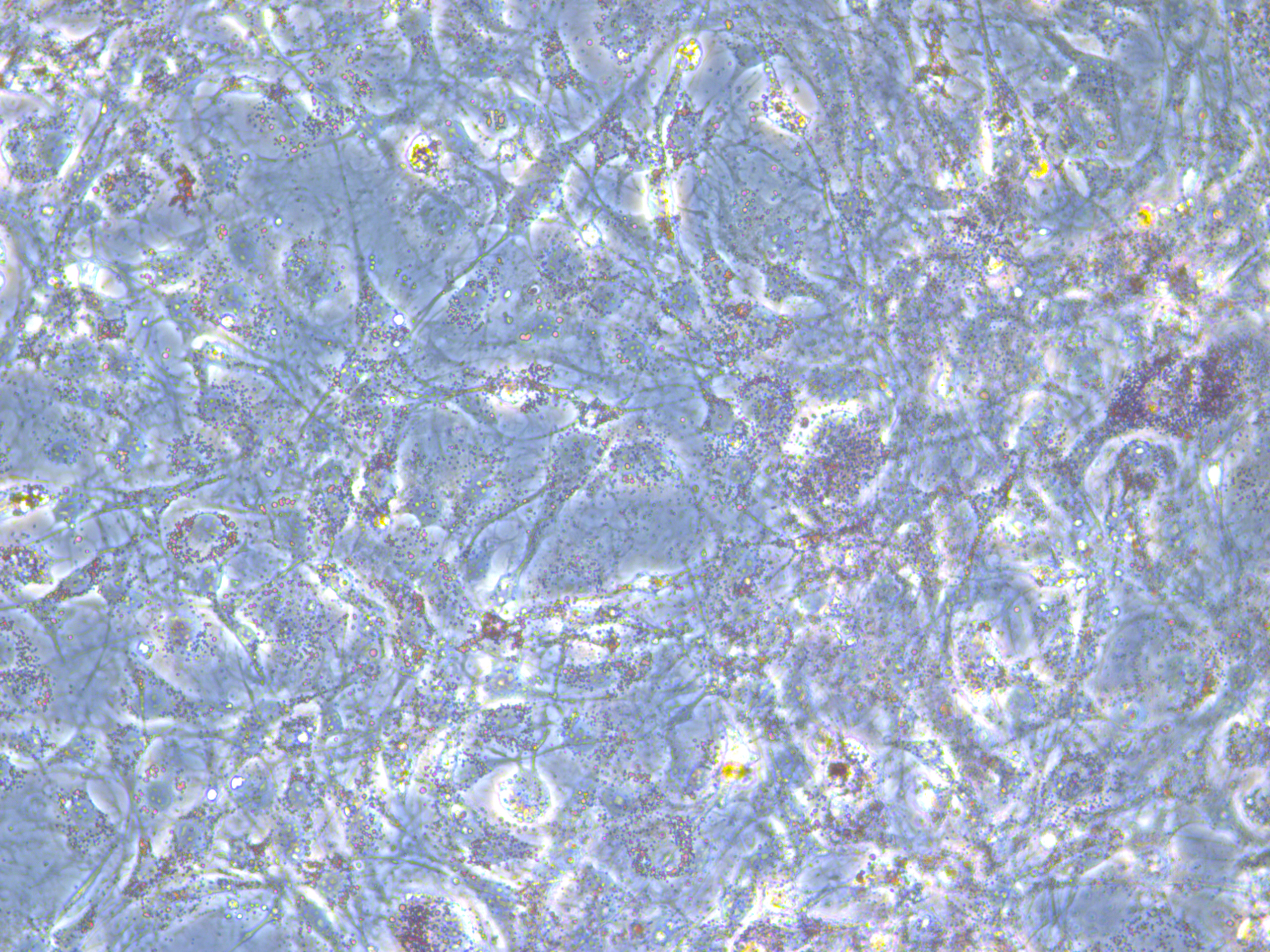

Supplement: Original Image for Fig 6c.jpg [file IENZ_A_2417915_SM7858.jpg]

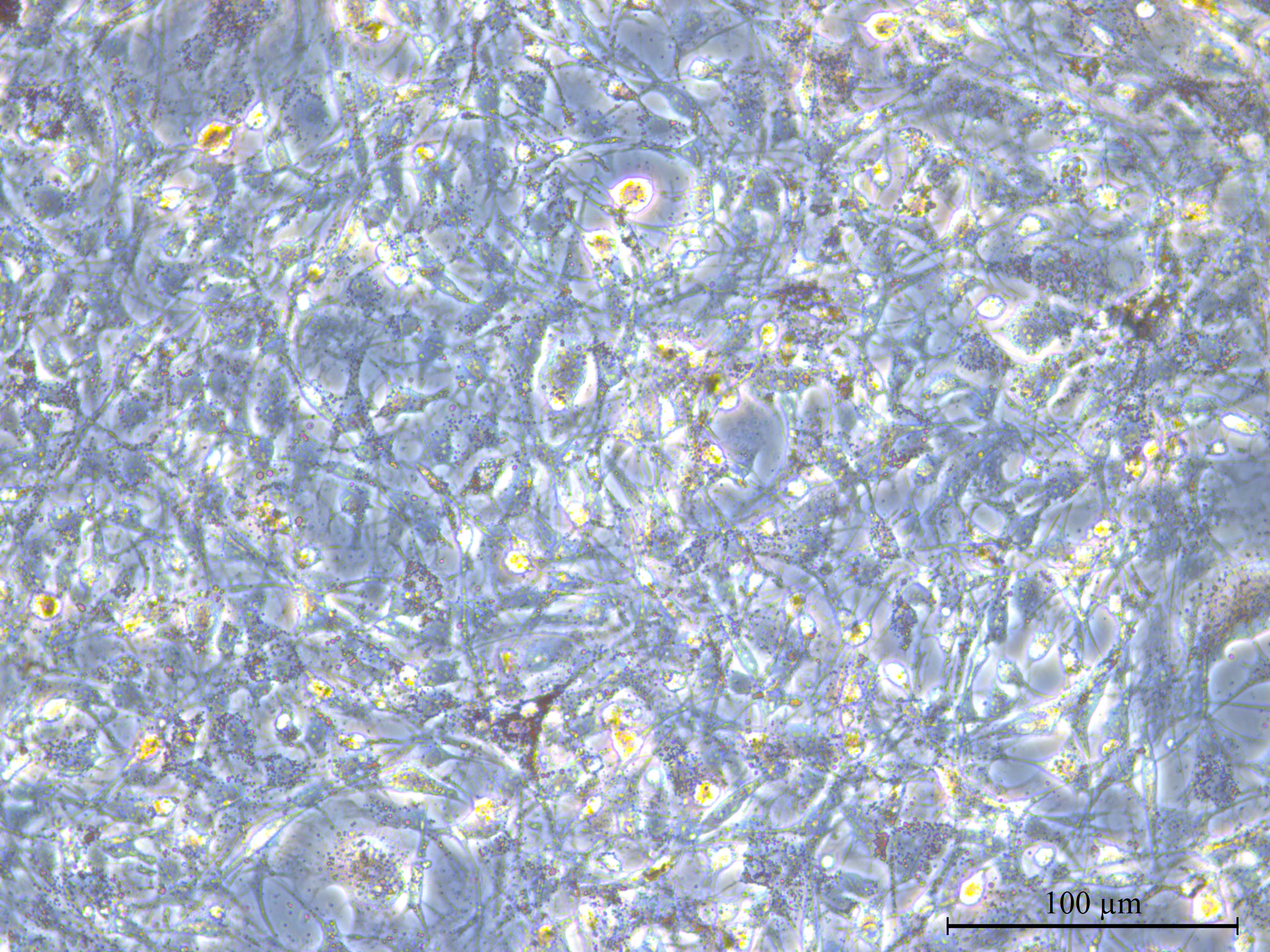

Supplement: Original Image for Fig 6e.jpg [file IENZ_A_2417915_SM7857.jpg]

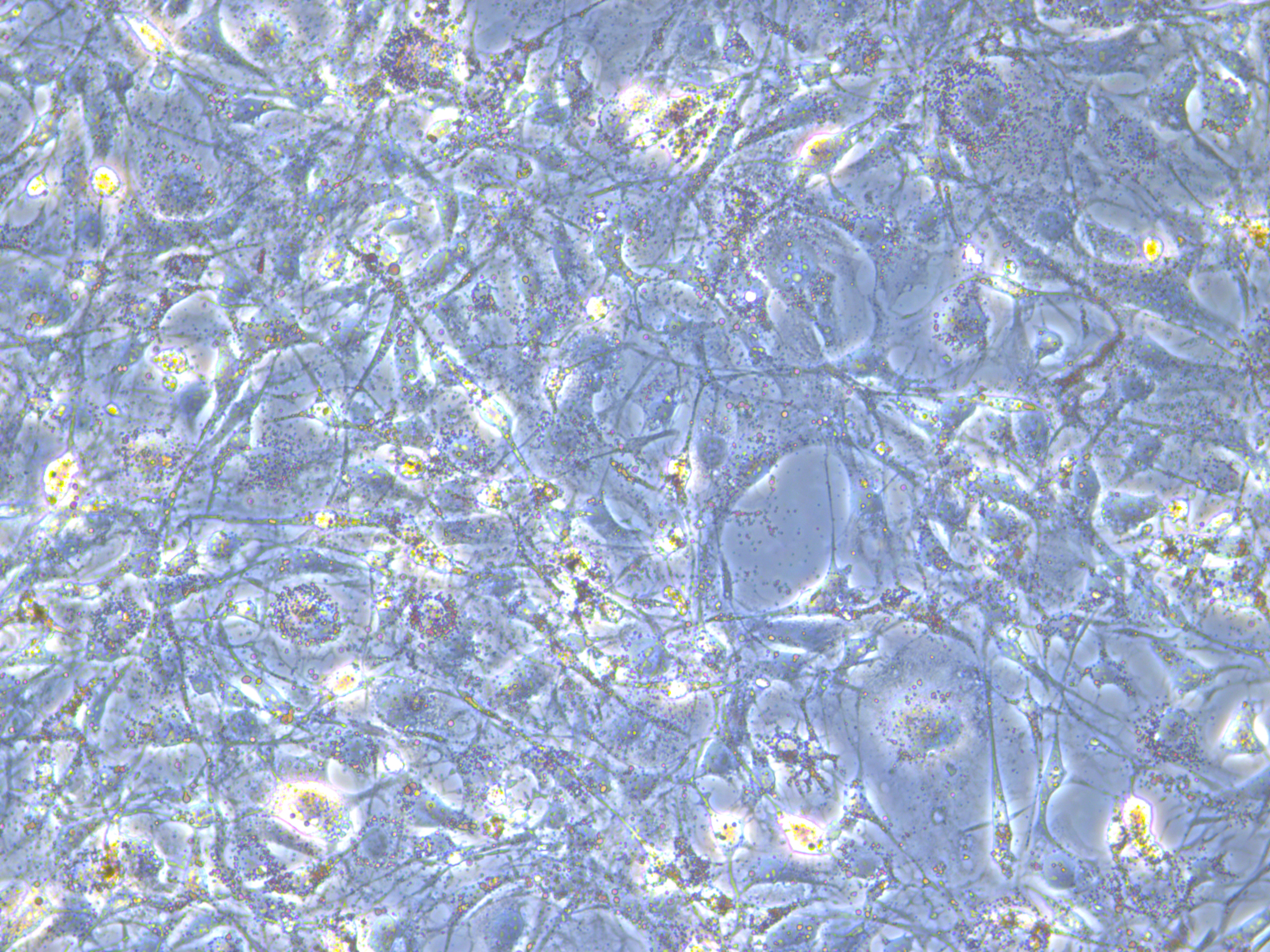

Supplement: Original Image for Fig 6d.jpg [file IENZ_A_2417915_SM7856.jpg]

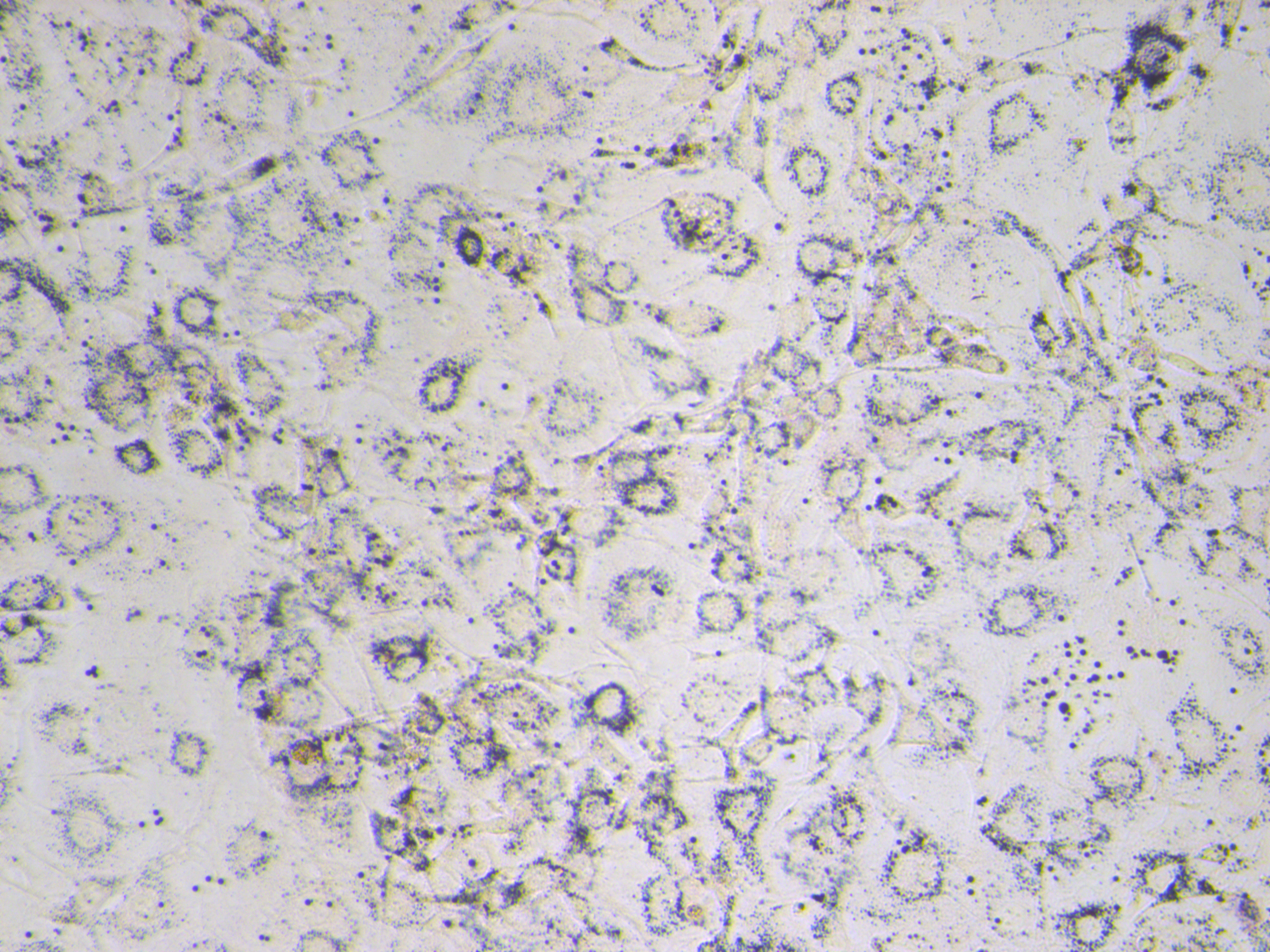

Supplement: Original Image for Fig 6a.jpg [file IENZ_A_2417915_SM7855.jpg]

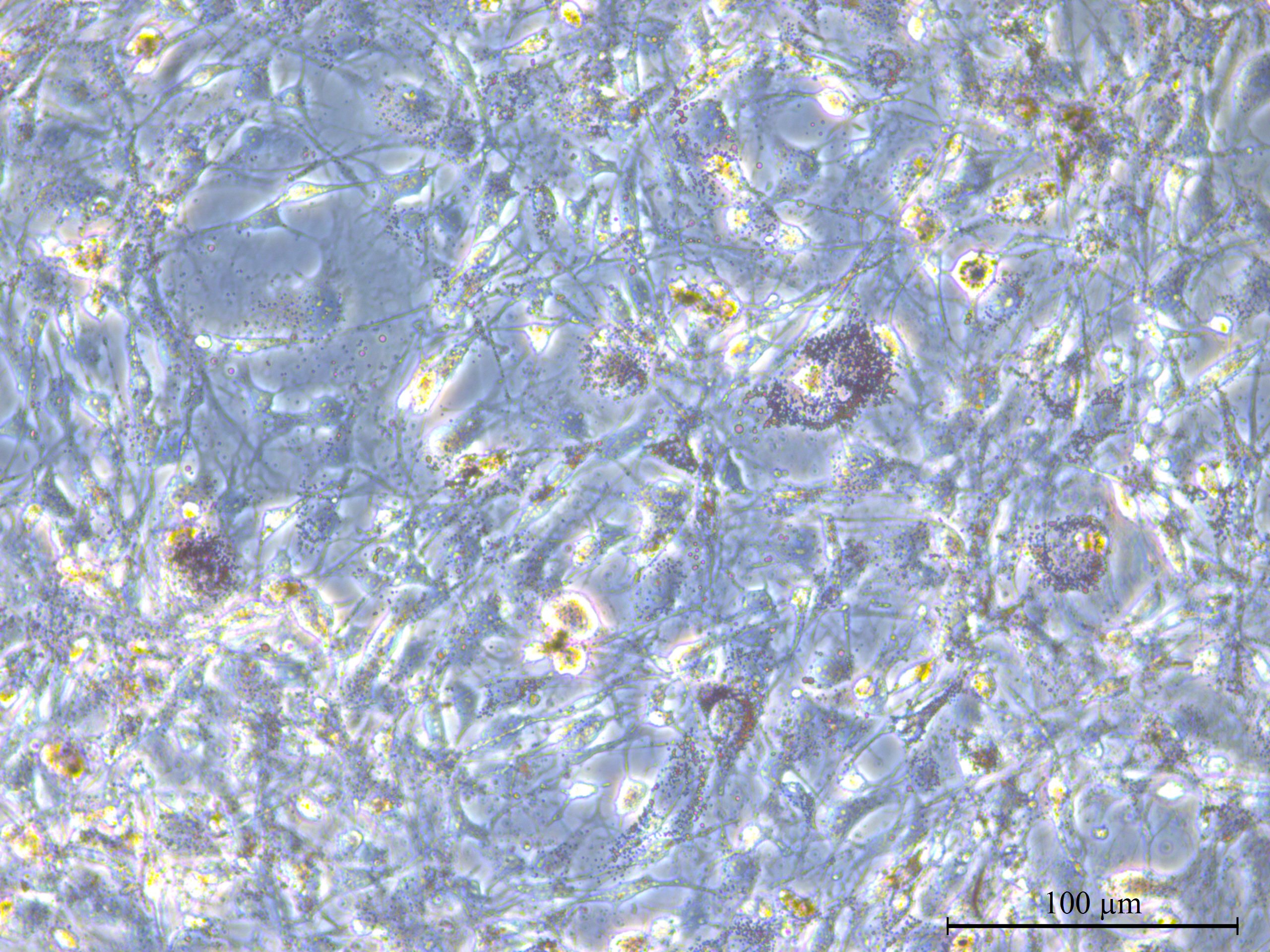

Supplement: Original Image for Fig 6g.jpg [file IENZ_A_2417915_SM7854.jpg]

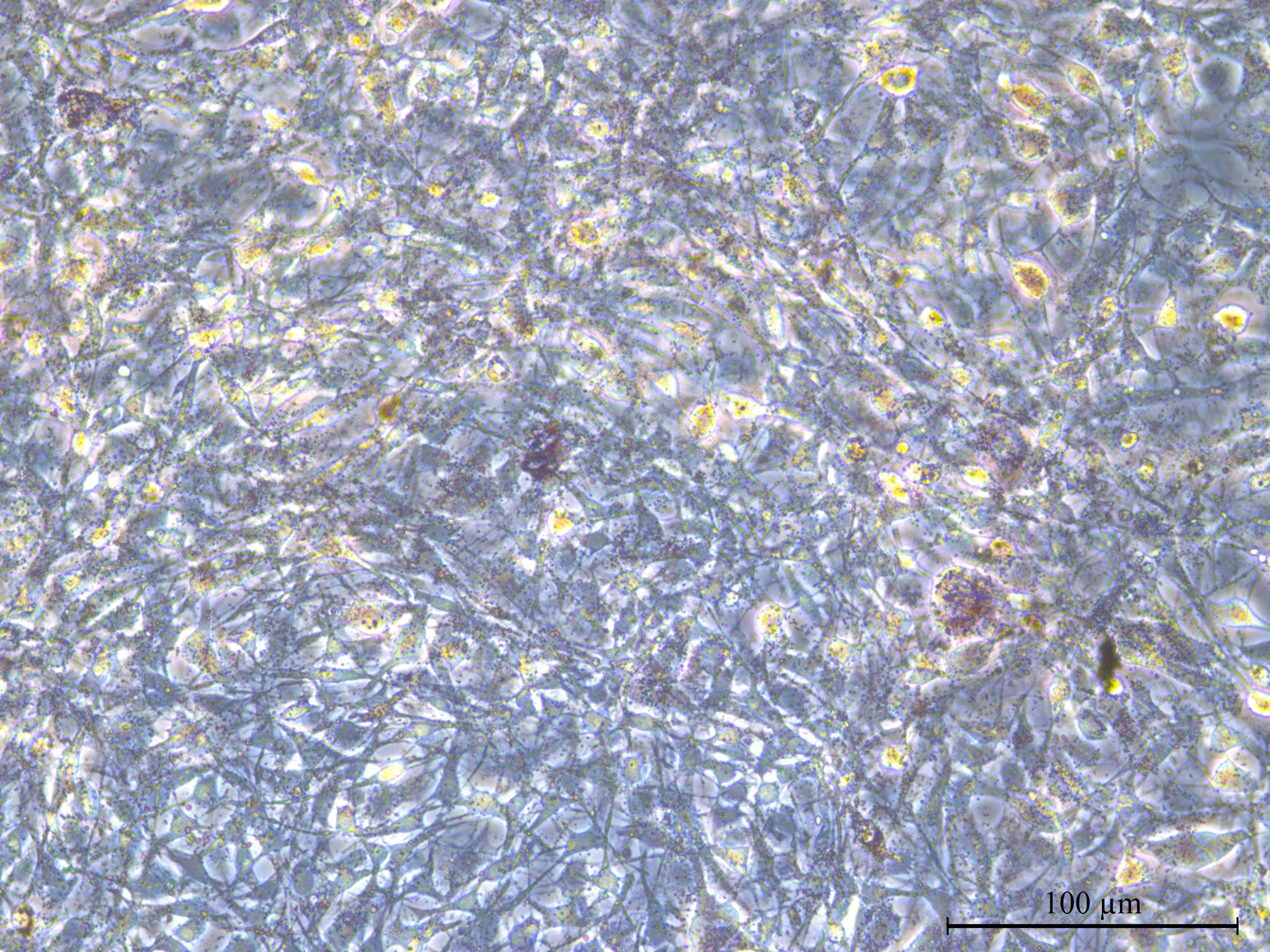

Supplement: Original Image for Fig 6f.jpg [file IENZ_A_2417915_SM7853.jpg]

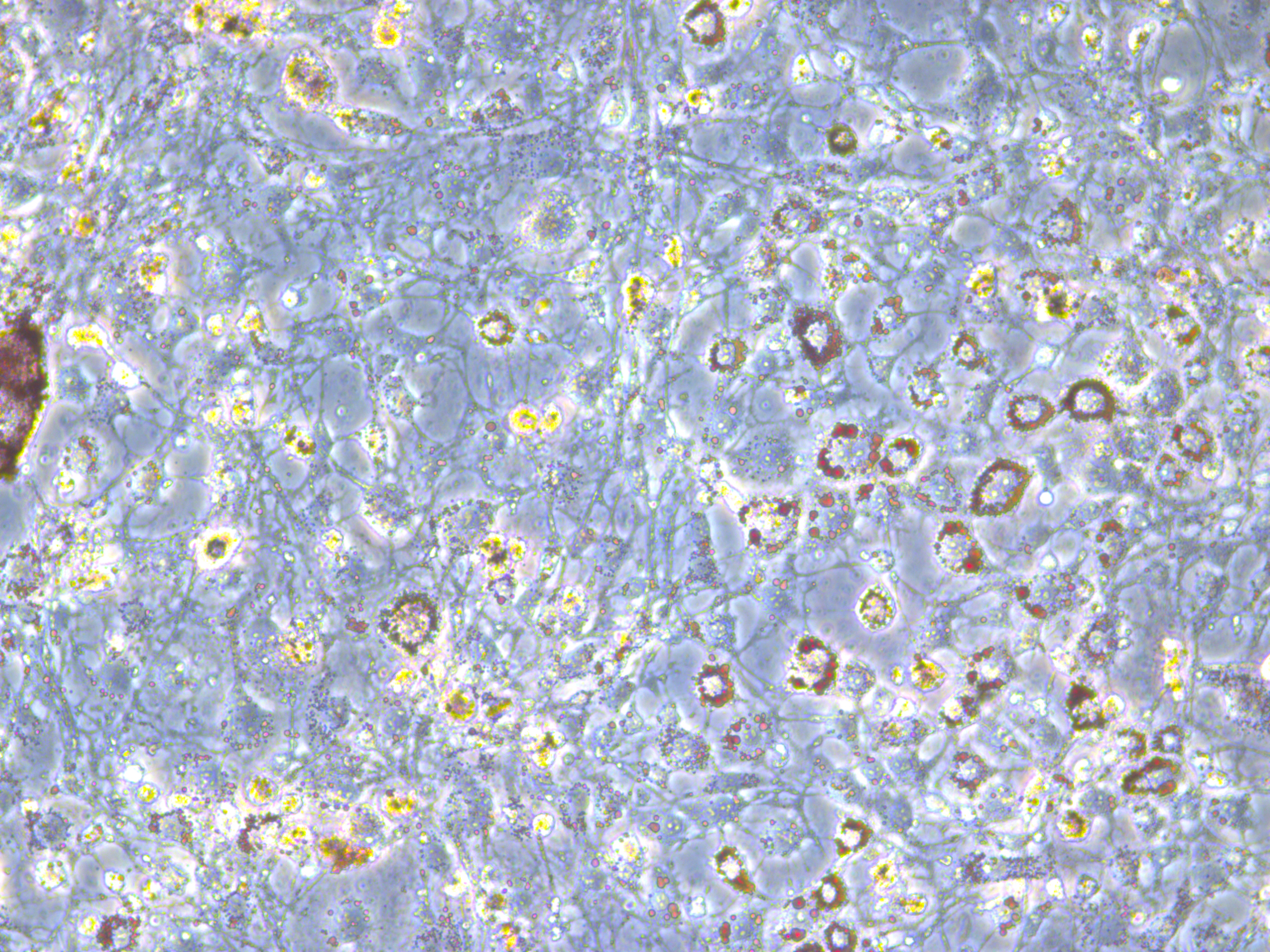

Supplement: Original Image for Fig 6b.jpg [file IENZ_A_2417915_SM7852.jpg]
